# Late effects and survival of children with malignant solid tumours in northern Finland: a single-centre cohort study

**DOI:** 10.1007/s00431-022-04399-7

**Published:** 2022-02-24

**Authors:** Sanni Kortelainen, Tekla Harju, Hanna Juntti, Tytti Pokka, Riitta Niinimäki

**Affiliations:** 1 PEDEGO Research Unit, University of Oulu, Oulu, Finland; 2 Department of Children and Adolescents, Oulu University Hospital, Oulu, Finland

**Keywords:** Late effects, Childhood, Solid tumours, Survival

## Abstract

The global survival rates for childhood cancers are high: approximately 80% of affected children will survive. Nevertheless, the burden of treatment for survivors is also high as three-quarters experience late effects of varying severity following cancer treatment. The aims of this study were to evaluate the treatment-related late effects of patients with childhood solid tumour in northern Finland and to report their survival rates. Our study included 104 patients treated for malignant solid tumours, excluding central nervous system tumours and lymphomas, between 1990 and 2015. Information regarding the type of late effects as well as other clinical data were obtained from the patients’ medical records. Late effects were observed in 65 (63%) patients, and almost half (40%) of the patients displayed more than one late effect. The most common late effect was hearing loss (*n* = 20). The 5-year survival rate in our study was 75%.

*   Conclusion*: Our results highlight the importance of long-term follow-up for childhood cancer survivors. As survivors age and survival rates improve, late effects and their impact on patient health as well as the value of surveillance must be considered.

**Table:** 

**What is Known:** *• Up to three-quarters of childhood cancer survivors experience treatment-related late effects.*	
**What is New:** *• The 5-year survival rate and the prevalence of late effects amongst childhood solid tumour patients treated in northern Finland are in line with findings from previous studies.*

**What is Known:**

*• Up to three-quarters of childhood cancer survivors experience treatment-related late effects.*

**What is New:**

*• The 5-year survival rate and the prevalence of late effects amongst childhood solid tumour patients treated in northern Finland are in line with findings from previous studies.*

## Introduction

Since the 1990s, about 80% of all childhood cancer patients in Finland have survived, representing one of the highest survival rates worldwide [1, 2]. As survival rates increase, treatment-related late effects cannot be ignored because of the improved life span for childhood cancer survivors [3, 4]. Late effects not only comprise physical and psychological challenges but also affect the overall quality of life in survivors [5].

Almost three-quarters of childhood cancer survivors experience late effects at some point following cancer treatment [3, 4]. The most commonly reported late effects are pulmonary, auditory and endocrine disorders [6]. Geenen et al. [4] reported that more than half of the survivors in their study had more than one adverse event simultaneously. Moreover, the frequency of late effects varies between tumour types. Nearly a third of Wilms tumour (WT) patients have been observed to have no adverse events [4], but bone tumour survivors are more likely to have multiple adverse events simultaneously [3, 4]. The severity of late effects also varies. For example, Wilms tumour patients are least likely to have severe or high-burden adverse events, whilst bone tumour survivors are more likely to experience these effects compared to other childhood cancer patients [3, 4].

Hudson et al. [6] estimated that almost all childhood cancer survivors will have a chronic condition by the age of 45 years, and Armstrong et al. [7] reported 54% of survivors having fatal, life-threatening, severe or disabling chronic conditions before 50 years of age. Compared to their siblings, childhood cancer survivors are 3.3 times more likely to have a chronic condition [3]. Given these numbers, it is extremely important to trace and assess possible late effects experienced by childhood cancer survivors. The aims of this study were therefore to establish the patient survival of childhood malignant solid tumour patients treated in a tertiary-level hospital in northern Finland and to evaluate the late effects experienced by this patient group.

## Material and methods

### Patients

**Fig. 1:**
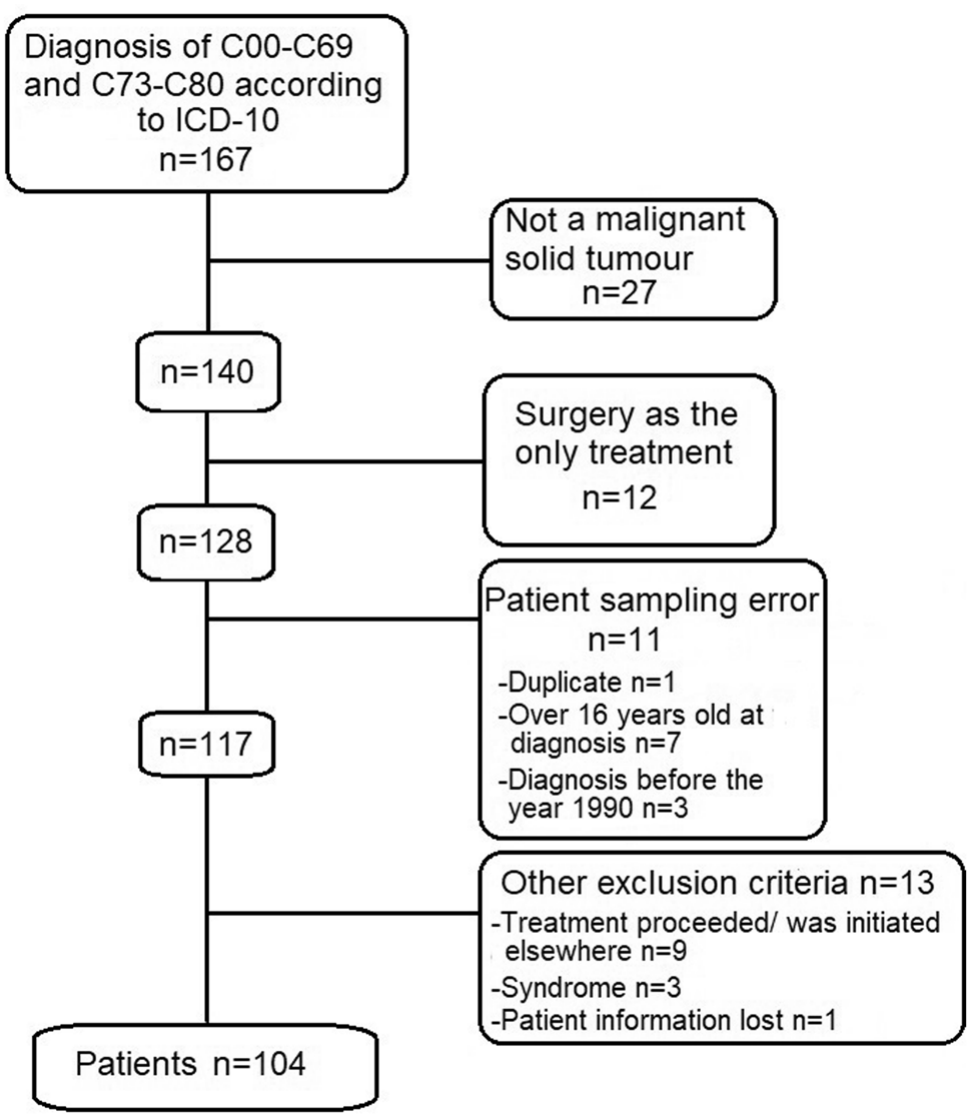
Flowchart of the patient selection. Not malignant solid tumour — category includes other childhood cancers that were first misdiagnosed as a solid tumour

This single-centre, retrospective cohort study was conducted in a tertiary-level hospital. The patients were identified from the hospital registry based on the diagnostic codes C00–C69 and C73–C80 of the 10th revision of the International Classification of Diseases (ICD–10) [8]. We wanted to focus on solid tumours excluding central nervous system tumours, because research on late effects in patients with brain tumours have been published previously [9, 10]. We also excluded lymphomas because of the similarity of their treatment to that of leukaemia. The inclusion criteria were patients who had been diagnosed between 1990 and 2015 and were younger than 16 years at the time of their diagnosis. The exclusion criteria and the patient selection process are shown in Fig. 1. We obtained all the data retrospectively, and there were no interventions involving the participants. Fertility and psychological late effects were excluded because they cannot be evaluated by only retrospective methods.

### Statistical analysis

We calculated the occurrence of late effects with 95% confidence intervals amongst the childhood cancer survivors and used the Kaplan–Meier method to evaluate the time to death for each patient following their respective cancer diagnoses. The differences in the cumulative survival between the different tumour types were assessed using a log-rank test. *P* value < 0.05 was considered statistically significant. We analysed the data using IBM SPSS Statistical Software for Windows version 27 (IBM Corp., released 2020, Armonk, NY).

## Results

**Fig. 2:**
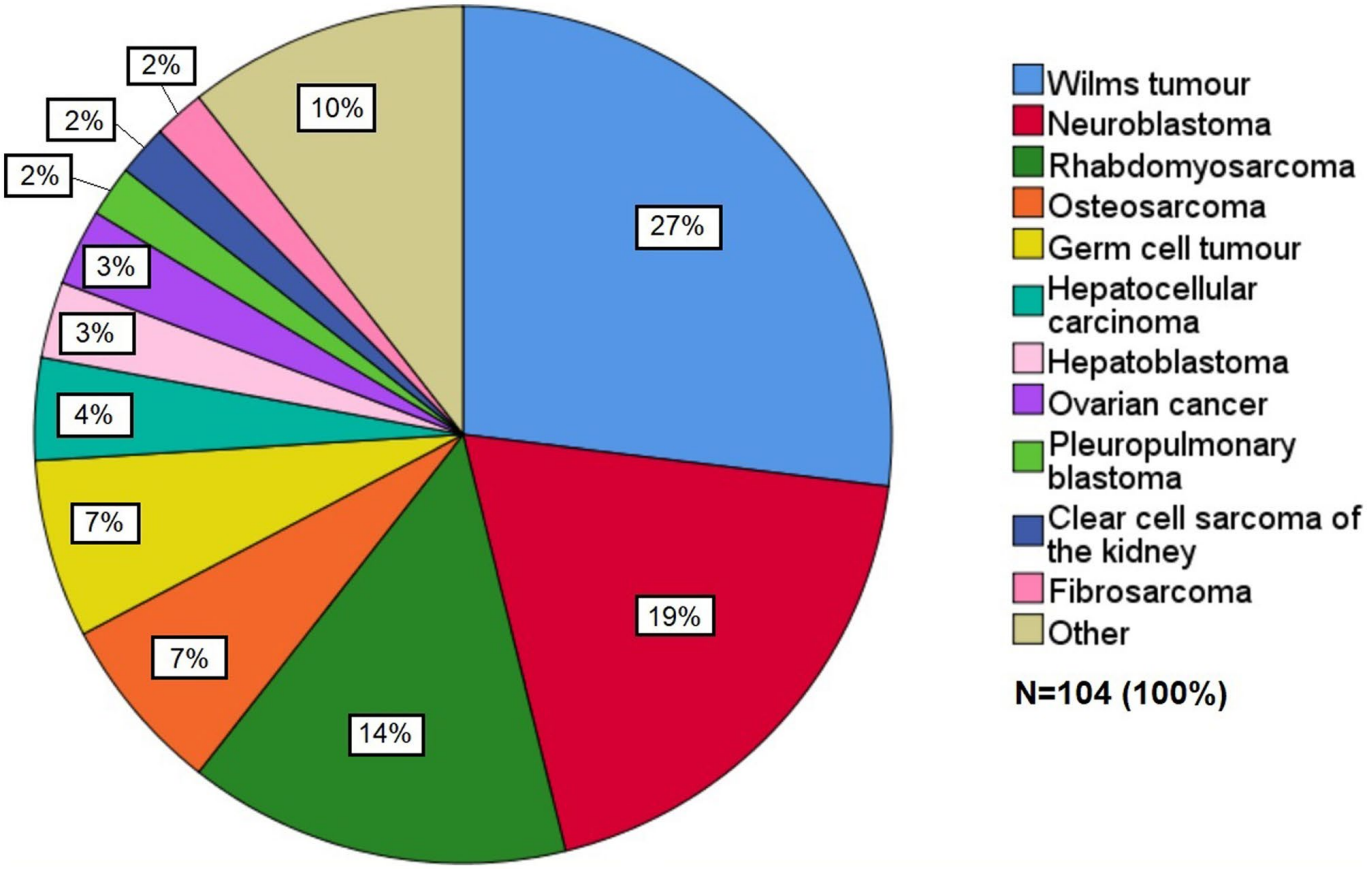
Diagnoses and their relative proportions of the solid tumours in the cohort. Other category includes the following solitary cancers: desmoplastic small-cell tumour, Ewing sarcoma, malignant peripheral nerve sheath tumour, melanocytic neuroectodermal tumour, melanoma, undifferentiated embryonal sarcoma of the liver, papillary thyroid carcinoma, peripheral neuroectodermal tumour, retinoblastoma, scrotal tumour and undifferentiated sarcoma

This study included 104 patients, of which 49 (47%) were male. The mean age at the time of diagnosis was 5.2 years (SD 4.5 years, range 0.01–15.7 years). The mean follow-up time was 10.2 years (range 0.1–28.1 years), and the median follow-up time was 9.4 years. Each patient who survived was followed up for at least 5 years. The diagnoses and their relative proportions are shown in Fig. 2.

### Late effects

In the cohort, 65 (63%) patients were observed to have one or more late effects following treatment. Multiple (two or more) late effects were noticed in 43 (40%) patients. In terms of the most common cancers (*N* ≥ 7) in the cohort, the highest number of late effects were noted in the patients with rhabdomyosarcoma (RMS) (11/15, 73%), followed by the patients with osteosarcoma (OS) (5/7, 71%). Multiple late effects were most common in the patients with OS (5/7, 71%) and those with RMS (9/15, 60%). The most common late effect was high-frequency hearing loss.

If not mentioned otherwise, the late effects were graded according to the Common Terminology Criteria for Adverse Events version 5.0 (CTCAE) [11], which grades late effects into five categories: grade 1, mild; grade 2, moderate; grade 3, severe; grade 4, life-threatening or urgent intervention indicated; and grade 5, death related to late effect (Table 1). Of the 65 patients with late effects, 13 had at least one heavy burden late effect (grade 3 or 4), whilst 48 patients had only moderate or mild (grade 1 or 2) late effects. Three patients suffered from death related to late effect; i.e. they had grade 5 late effect. For one patient with ovarian failure, the late effect was graded differently.

**Table 1:** The number of observed late effects and their percentage values in the cohort, *N* = 104

**Late effect**	***N* = 104, *n* (%)**	**95% CI**	**Grade 1**	**Grade 2**	**Grade 3**	**Grade 4 or 5**	**Primary tumour (*n*)**
Sensory system morbidity	23 (22.1)	[14.6–31.3]					
Hearing loss	20 (19.2)	[12.2–28.1]	7	3	6	2	NB (6), OS (4), HB (3), GCT (2), HCC (2), RMS (2), MNTI (1)
Decreased vision or cataract	3 (2.9)	[0.6–8.2]	-	1	1	1	RMS (2), WT (1)
Osteoporosis or osteopenia	7 (6.7)	[2.7–13.4]	3	4	-	-	OS (4), WT (2), NB (1)
Other bone morbidity	4 (3.8)	[1.1–9.6]	2	1	1	-	NB (1), PPB (1), RMS (1), WT (1)
Other musculoskeletal morbidity	22 (21.2)	[13.8–30.3]					
Scoliosis	9 (8.7)	[4.0–15.8]	4	3	2	-	NB (2), OS (2), WT (2), RMS (1), CCSK (1), PPB (1)
Achilles tendon tension	5 (4.8)	[1.6–10.9]	5	-	-	-	NB (2), FS (1), WT (1), RMS (1)
Amputation or prosthesis	4 (3.8)	[1.1–9.6]	-	4	-	-	OS (4)
Muscle atrophy or weakness	4 (3.8)	[1.1–9.6]	-	4	-	-	RMS (1), PNET (1), OS (1), US (1)
Neurological defect	15 (14.4)	[8.3–22.7]					
Neuropathy	5 (4.8)	[1.6–10.9]	-	5	-	-	RMS (2), GCT (1), HB (1), WT (1)
Other neurological defect	7 (6.7)	[2.7–13.4]	3	-	4	-	NB (3), HB (2), OS (1), RMS (1)
Antiepileptic medication	3 (2.9)	[0.6–8.2]	3	-	-	-	GCT (1), NB (1), RMS (1)
Benign tumour	10 (9.6)	[4.7–17.0]	5	5	-	-	RMS (3), WT (3), GCT (2), NB (1), OS (1),
Tumour-like lesion	20 (19.2)	[12.2–28.1]	20	-	-	-	RMS (5), WT (4), NB (2), FS (2), CCSK (1), ES (1), GCT (1), HCC (1), OS (1), PTC (1), PPB (1)
Second cancer	4 (3.8)	[1.1–9.6]	-	-	1	3	HB (1), RB (1), RMS (1), WT (1)
Cardiovascular symptoms	6 (5.8)	[2.1–12.1]	3	1	2	-	GCT (1), HB (1), MPNST (1), NB (1), OS (1), PNET (1)
Anaemia	4 (3.8)	[1.1–9.6]	4	-	-	-	WT (3), HCC (1)
Respiratory symptoms	7 (6.7)	[2.7–13.4]	-	7	-	-	NB (3), WT (3), RMS (1)
Urinary and kidney defect	14 (13.5)	[7.6–21.6]					
Kidney dysfunction	7 (6.7)	[2.7–13.4]	4	2	-	-	NB (3), OS (2), GCT (1), RMS (1)
Incontinence	4 (3.8)	[1.1–9.6]	3	1	-	-	NB (2), CCSK (1), GCT (1)
Other urinary defect	3 (2.9)	[0.6–8.2]	1	1	1	-	RMS (2), NB (1)
Digestive tract defect	17 (16.3)	[9.8–24.9]					
Dental problem	7 (6.7)	[2.7–13.4]	5	2	-	-	NB (2), RMS (2), WT (2), HB (1)
Other digestive tract defect	6 (5.8)	[2.1–12.1]	1	1	1	-	CCSK (1), FS (1), GCT (1), HB (1), RMS (1), WT (1)
Problems with defecation	4 (3.8)	[1.1–9.6]	2	2	-	-	NB (3), RMS (1)
Endocrine dysfunction	15 (14.4)	[8.3–22.7]					
Testicular failure	6 (5.8)	[2.1–12.1]	-	-	-	-	RMS (3), NB (2), HB (1)
Ovarian failure	5 (4.8)	[1.6–10.9]	-	-	-	-	WT (2), CCSK (1), NB (1), OS (1)
Growth hormone deficiency	4 (3.8)	[1.1–9.6]	-	4	-	-	ES (1), HCC (1), NB (1), RMS (1)

Late effects have been graded according to the CTCAE version 5.0

*NB* neuroblastoma, *OS* osteosarcoma, *HB* hepatoblastoma, *GCT* germ cell tumour, *HCC* hepatocellular carcinoma, *RMS* rhabdomyosarcoma, *MNTI* melanocytic neuroectodermal tumour, *WT* Wilms tumour, *FS* fibrosarcoma, *CCSK* clear cell sarcoma of the kidney, *ES* Ewing sarcoma, *PTC* papillary thyroid carcinoma, *PPB* pleuropulmonary blastoma, *RB* retinoblastoma, *PNET* peripheral neuroectodermal tumour, *US* undifferentiated sarcoma, *MPNST* malignant pheripheral nerve sheat tumour

#### Sensory system

The most common sensory system morbidity was high-frequency hearing loss, which was observed in 18 patients (Table 1). Two patients had sensorineural hearing loss classified as grade 4. For two patients audiograms were not available for grading. Four patients needed a hearing aid or hearing aids. Decreased vision or cataract was detected in two patients due to irradiation near the symptomatic eye. A third patient with decreased vision experienced other neurological late effects such as vincristine neuropathy and strabismus. The cataract case was graded as grade 4, and decreased vision cases (*n* = 2) were grade 3 and 2.

#### Bone and musculoskeletal system

Bone morbidity was observed in 11 (11%) patients, and 22 (21%) patients had other musculoskeletal morbidities (Table 1). The patients with OS were the most represented patient group amongst those with osteoporosis or osteopenia (*n* = 6), and all the patients who survived OS had either a prosthesis, amputation or allogenic bone graft. Prosthesis and amputations were classified as musculoskeletal deformities according to the CTCAE. Three of the osteoporosis cases were grade 1, which are considered osteopenia. Scoliosis was diagnosed in nine (9%) patients. Two (2%) patients had grade 4 scoliosis; they both required corset treatment, whilst one also had surgery. The patients who suffered from scoliosis also had other late effects, including paraparesis, prosthesis, osteopenia or osteoporosis, chondromalacia, muscle weakness, length difference and faecal and urinary incontinence.

Other bone morbidities were osteonecrosis (*n* = 1), chondromalacia (*n* = 1), hyperlordosis (*n* = 1) and bone developmental disorder (*n* = 1). Local irradiation led to osteonecrosis and hyperlordosis in two patients, and bone developmental disorder in one patient. The bone developmental disorder was grade 3, and it was classified according to musculoskeletal deformity category of the CTCAE. Chondromalacia was graded according to its symptoms (arthralgia) as grade 1. Three patients with muscle atrophy had lower extremity primary tumours and had received irradiation in the area of the atrophy. One patient had muscle weakness in the lower extremity near the primary tumour. Muscle atrophies were also classified based on musculoskeletal deformities according to the CTCAE, whilst Achilles tendon tensions were classified as “decreased joint range of motion.”

#### Neurology

The most common neurological defect was neuropathy (*n* = 5; Table 1). Four patients had received vincristine and two cisplatin. Other neurological defects were encephalopathy (*n* = 2), idiopathic headache (*n* = 2), paraparesis (*n* = 2) and working memory impairment (*n* = 1). Encephalopathy was diagnosed via magnetic resonance imaging in one patient after stem cell transplantation (SCT). Moreover, in another patient who had ifosfamide-related encephalopathy, changes were seen on electroencephalography. The patient with ifosfamide-related encephalopathy had received methylene blue under ifosfamide treatment. Both encephalopathies were grade 3. The paraparesis cases, classified based on the spinal cord compression category of the CTCAE, were grade 3. Three patients required antiepileptic medication due to seizures.

#### Endocrine dysfunction

The incidence of testicular failure amongst the male patients (*n* = 49) was 12% (Table 1). Five patients who had primary testicular failure had received alkylating agents, whilst high-dose (HD) chemotherapy with autologous SCT had been given to the patients with neuroblastoma (NB). Only one patient suffered from both primary and secondary hypogonadism, possibly caused by cranial irradiation in combination with the alkylating agents. In this study, testicular failure was defined by the absence of puberty or small post-pubertal testicle size with a history of increased luteinizing hormone and follicle-stimulating hormone levels along with low testosterone levels.

The incidence of ovarian failure amongst the females patients (*n* = 55) was 9%. Except for one patient, all of these patients had received abdominal irradiation and cyclophosphamide. One patient had also received HD chemotherapy with autologous SCT. Ovarian failure was defined by the absence of puberty or a history with increased luteinizing hormone and follicle-stimulating hormone, low oestrogen and anti-Mullerian hormone levels and an abnormally small number of follicles seen via ultrasound. Amongst the patients with growth hormone deficiency, one had received cranial irradiation, whilst two patients had received HD chemotherapy with autologous SCT. One patient had no evident predisposing factors for growth hormone deficiency.

#### Second cancer related to treatment

One patient who was diagnosed with a gastro-intestinal clear cell tumour and one patient who was diagnosed with glioblastoma as second tumours had received irradiation to the location where the second malignant tumour had developed (Table 1). One patient subsequently developed acute myeloid leukaemia. These three patients did not have any cancer predisposing syndromes and their second cancers were classified as grade 5 (death related to late effect). One patient who was diagnosed with low-grade glioma as a second tumour had received irradiation to the location where the second tumour had developed. This patient had a cancer-predisposing gene mutation, and his second tumour was grade 3.

#### Cardiovascular and respiratory symptoms

Cardiovascular symptoms were observed in six (6%) patients (Table 1), and included hypertension (*n* = 2), lymphoedema (*n* = 2), heart failure (*n* = 1) and thrombus (*n* = 1). Both hypertension cases were grade 3. The primary tumours of both patients diagnosed with lymphoedema had been located in the thigh, and these patients suffered from swelling in this lower extremity. The heart failure case was grade 1, and the thrombus case was grade 2. One patient had normocytic and normochromic anaemia, which was possibly caused by immunosuppressive medication, whilst the other three patients with anaemias had iron-deficiency anaemias.

Most of the respiratory symptoms were due to asthma (*n* = 6; Table 1). Four of the six patients with asthma had been treated with anthracyclines, and five of the six patients had received cyclophosphamide. One patient had impaired pulmonary function, and she had received lung irradiation, and a thoracotomy had also been performed due to lung metastases. The pulmonary function impairment was grade 2 when classified according to the vital capacity grading of the CTCAE.

#### Urinary and kidney defects

Seven (7%) patients were diagnosed with kidney dysfunction, of which six were diagnosed during treatment (Table 1). The kidney dysfunctions were classified according to “chronic kidney disease” in the CTCAE except for one patient for whom the classification was not applicable due to missing exact data regarding their kidney parameters. Only for one patient the kidney function improved during follow-up, whilst for the others it remained poor.

The other urinary defect group consisted of patients with neurogenic bladder (*n* = 1), urinary tract obstruction (*n* = 1) and urinary retention (*n* = 1). The patients with neurogenic bladder and urinary tract obstruction also developed kidney dysfunction. Urinary tract obstruction was considered grade 3. Neurogenic bladder was considered grade 2 when classified according to its symptom (urinary incontinence) of the CTCAE. Three patients were suspected to have functional incontinence, and, in one patient, their urinary incontinence was grade 2 with a neurological aetiology.

#### Digestive tract

Digestive tract late effects were observed in 17 (16%) patients (Table 1). The most common dental problem was a missing permanent tooth or teeth (*n* = 5). A further dental problem was hypomineralisation (*n* = 2). The dental problems were classified as tooth development disorders based on the CTCAE.

Two of the defaecation problems were constipation, and two were incontinence. The cause of defaecation incontinence was neurological for one patient and functional for the other, and these defects were considered to be grade 1. Other digestive tract defects were biloma (*n* = 1), cholangitis (*n* = 1), permanent ostomy (*n* = 1), gastro-oesophageal reflux (*n* = 1), diarrhoea (*n* = 1) and bowel obstruction (*n* = 1). Diarrhoea was grade 1, gastro-oesophageal reflux was grade 2 and bowel obstruction was grade 3. Grading could not be performed for the biloma, cholangitis or permanent ostomy cases due to a lack of suitable criteria in the CTCAE.

#### Benign tumours and tumour-like lesions

After the primary tumour, a single benign tumour was found in nine patients, and five of these patients had received irradiation (Table 1). Only one patient had more than one benign tumour. Examples of benign tumours were fibroadenoma, angiomyolipoma and haemangioma. Most of the tumour-like lesions were ovarian cysts (*n* = 4) and hypervascular lesions in the liver (*n* = 4; Table 1). The other lesions included ectopic tissue and cysts in organs other than the ovaries. These lesions were diagnosed by imaging, and a biopsy was not always required.

### Survival

**Fig. 3:**
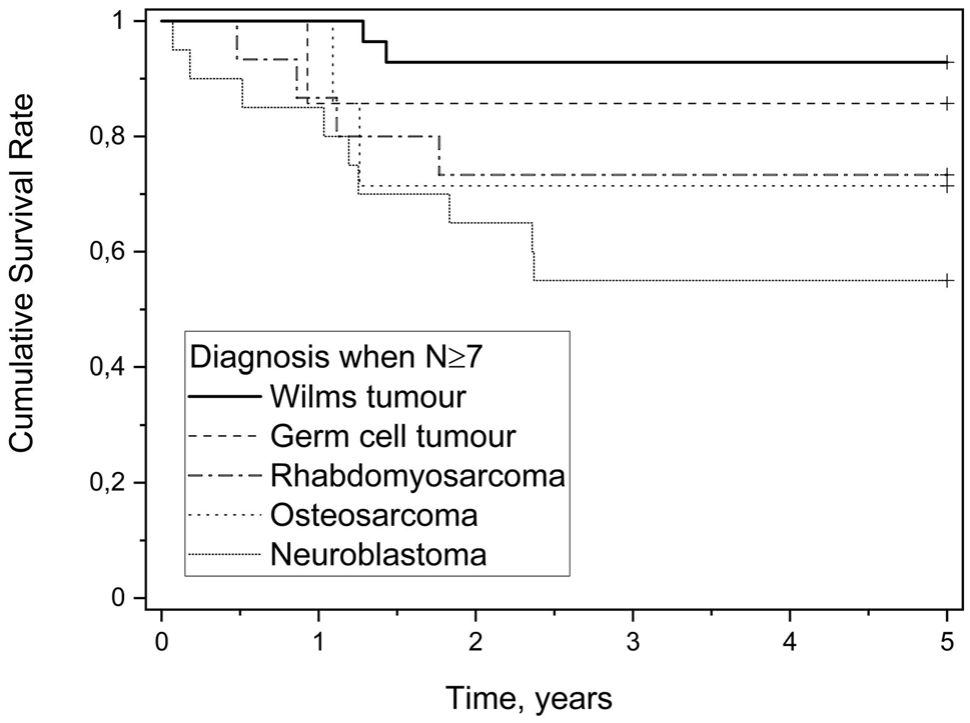
Kaplan–Meier curve showing survival rates of the most common tumours in this study (*N* ≥ 7)

In the cohort of 104 patients, 31 (30%) were deceased by end of the study, and the 5-year survival rate was 75%. Amongst the deceased patients 10 (32%) died in the first year following diagnosis, and only five patients lived more than 5 years following diagnosis. Amongst the most common tumours (*N* ≥ 7) in this study, the highest survival rate was seen in the patients with WT and the lowest in those with NB, as shown in Fig. 3. We observed differences in survival amongst the most common diagnosis groups (log-rank test, *p* = 0.039).

## Discussion

In this study, 63% of the childhood cancer survivors had at least one late effect, whilst multiple late effects were observed in 40% of the patients. Three (3%) patients suffered from death related to late effect, and 13 (13%) had at least one heavy burden late effect (grade 3 or 4) potentially affecting their quality of life. The most common late effect was hearing loss. Bone and musculoskeletal defects, which affected 32% of the patients, comprised the largest grouped late effect entity, and neuroendocrine dysfunction was observed in one third (30%) of the patients.

The prevalence of late effects in our study was similar to that of previous studies [3, 4]. In our cohort, all patients with hearing loss had been treated with cisplatin during chemotherapy except for one patient who had received cranial irradiation, which could have been a predisposing factor. Bertolini et al. [12] studied childhood patients diagnosed with NB, OS, hepatoblastoma and germ cell tumour and reported that hearing loss was most often detected when the dose of cisplatin was at least 400 mg/m^2^ [12]. Peleva et al. [13] noted that 48% of the patients in their study had ototoxicity after treatment with platin compound chemotherapy for childhood solid tumours. These results are compatible with those of our study.

Musculoskeletal morbidities were most common in the patients with OS in our cohort. Considering the need for amputations and prostheses, it is not surprising that compared to other childhood cancer patients and healthy siblings, patients with OS are more likely to have life-long functional limitations [14, 15]. Interestingly, patients with OS have not reported major emotional problems compared to other childhood cancer patients [16], and their self-reported functional status is better than could be expected [14]. Eiser et al. [16] suggested that involving patients in the decision-making with regard to amputation or limb salvage surgery may lead to better long-term psychological outcomes. As reported in earlier studies [3, 4], we observed that multiple late effects were most common in the survivors of OS. Aside from musculoskeletal late effects, these patients in our cohort developed sensory system, cardiovascular and urinary tract morbidities which could be related to their treatment with multimodal chemotherapy [4].

Spinal malalignment after cancer treatment is associated with asymmetrical irradiation [17]. Furthermore, the paraspinal tumours can cause spinal cord or column compression leading to the postural anomalies that are often seen in patients with NB [18]. In our cohort, two patients with NB had scoliosis because of the location of their primary tumours, and two patients with OS had scoliosis as a result of their prosthesis or bone craft. The other five patients with scoliosis had no evident predisposing factors, but four received asymmetrical irradiation to the trunk.

Neuropathies are closely interrelated with musculoskeletal function [19]. In our cohort, most of the neuropathies manifested as a motor impairment, which presented as clumsiness. Only two patients had sensory symptoms, such as dysaesthesia related to neuropathy. The patients with neuropathies had received either or both vincristine and cisplatin, which are the most common neurotoxic cytotoxins [19].

Alkylating agents, irradiation and bone marrow transplants are well-known risk factors for endocrinological late effects [20–23]. Many of our patients with testicular or ovarian failure received these treatments. Gonadal failure has a large impact on patient quality of life [5] as it not only cause infertility, but also predisposes patients to, for example osteoporosis, cardiovascular diseases, impaired sexual function and delayed puberty [20, 22]. Hormonal changes alone do not predict fertility because of the possible discordant correlation between these two parameters [24].

Second tumours are usually seen as one of the most severe late effects of cancers and can occur even decades after the first cancer. Treatment with both chemotherapy and irradiation places patients at an increased risk of developing a second malignancy [25]. Patients with RMS reportedly have the highest relative risk for a second malignant neoplasm compared to those with other childhood cancers [25]. In our cohort, three out of the four patients with a second malignancy had received treatment with both chemotherapy and irradiation.

The prevalence of incidentalomas varies from 5 to 30% depending on the imaging model [26]. The high number of tumour-like lesions (*n* = 20) and benign tumours (*n* = 10) in our cohort can be partly accounted for by the imaging that is part of the surveillance for childhood cancer patients. These meaningless findings may be discovered unintentionally, but they can represent a psychological ordeal for the patient. Thompson et al. [27] reported that 37% of the long-term lymphoma survivors in their study experienced significant anxiety with regard to surveillance scans, but a better doctor-patient relationship was associated with lower levels of anxiety.

Anthracycline [28] and cyclophosphamide [29] can place patients at an increased risk for chronic cough. In our cohort, every patient diagnosed with asthma had received either or both of these cytotoxins as part of their treatment, and chronic cough was a common symptom prior to their asthma diagnosis. Other reported predisposing factors to pulmonary late effects are asparaginase, bleomycin, lung irradiation and thoracotomy [6, 28, 29].

The prevalence of hypertension amongst childhood cancer survivors has been reported by previous studies to be higher than that of the general population, and the prevalence has been shown to increase with age [20]. Gibson et al. [30] reported that many childhood cancer survivors had uncontrolled or undiagnosed hypertension when enrolling in their study. The lack of surveillance in adulthood could partly explain the low prevalence of hypertension in our cohort. Heart irradiation, anthracyclines and female sex are risk factors associated with heart failure [31]. Our patient with heart failure met these criteria by being female and having been treated with anthracyclines. Hudson et al. [6] reported an abnormal blood count in 3% of the childhood cancer survivors treated with alkylating agents, anthracyclines and epipodophyllotoxin chemotherapy in their study. This prevalence was comparable with that in our study (4%). Moreover, all the patients in our study who developed anaemia had also been treated with anthracycline.

In Europe, the 5-year survival for all childhood cancer patients (age 0–14 years) was 71.8% [32] and 77.9% [33] in 1990–1994 and 1999–2007, respectively, and the overall survival in our cohort was comparable to these survival rates. The 5-year survival rates of the patients with WT and OS were the same or higher in our cohort than the concomitant average European survival rates [32, 33]. In contrast, the survival rates of the NB and RMS patients were considerably lower in our cohort [32, 33]. In the USA, the 5-year survival for all childhood cancer patients (age 0–14 years) was 75.8% and 83.8% in 1990–1992 and 2007–2014, respectively [34]. The most significant difference between our study findings and those in the USA was seen in the patients with NB as the 5-year survival rate for NB patients in 1990–2014 remained consistently above 70% in the USA [34], whereas in Finland, it has never been as high [2]. The 5-year survival rate for the WT patients in our cohort (89.3%) was very similar to that of the USA (89.4% in 2003–2007) [34].

This study highlights the importance of surveillance for childhood cancer patients. The screening of late effects should be taken into consideration when treating childhood cancer patients as this patient group has a potential full lifetime ahead of them. The development of models for risk prediction is the prevailing practice in many countries, but new models should take into account the cost-effectiveness of surveillance and quality of life [35]. It is not reasonable to expose patients to expensive screening modalities when the risks of predicted late effects are low, particularly if the patient feels that long-term screening may contribute to a reduced quality of life [35]. A gold standard for the surveillance of childhood cancer patients is still under development, and further research into the late effects of childhood cancer and their characteristics is thus required.

## Data Availability

The data that support the findings of this study are available on request from the corresponding author.

## Abbreviations

CTCAE: Common Terminology Criteria for Adverse Events
HD: High dose
NB: Neuroblastoma
OS: Osteosarcoma
RMS: Rhabdomyosarcoma
SCT: Stem cell transplantation
WT: Wilms tumour
